# Hepatitis E virus neutralization by porcine serum antibodies

**DOI:** 10.1128/jcm.00373-23

**Published:** 2023-10-12

**Authors:** Nele Gremmel, Oliver Keuling, Martin Eiden, Martin H. Groschup, Reimar Johne, Paul Becher, Christine Baechlein

**Affiliations:** 1 Department of Infectious Diseases, Institute of Virology, University of Veterinary Medicine, Hannover, Germany; 2 Institute for Terrestrial and Aquatic Wildlife Research, University of Veterinary Medicine, Hannover, Germany; 3 Institute of Novel and Emerging Infectious Diseases, Friedrich-Loeffler-Institut, Federal Research Institute for Animal Health, Greifswald, Insel Riems, Germany; 4 Department of Biological Safety, German Federal Institute for Risk Assessment, Berlin, Germany; Boston Children's Hospital, Boston, Massachusetts, USA

**Keywords:** hepatitis E virus, immune response, neutralizing antibodies, assay development

## Abstract

The consumption of raw or undercooked meat products poses a serious risk for human hepatitis E virus (HEV) infections. In many high-income countries, domestic pigs and wild boars represent the main animal reservoirs for HEV and are usually identified by reverse transcription-PCR and antibody enzyme-linked immunosorbent assay (ELISA). In order to characterize the humoral immune response in more detail, a cell culture-based serum neutralization assay using a culture-adapted HEV strain was established here. Measurement of neutralizing antibodies was only possible after removing the viral quasi-envelope by detergent treatment. Serum samples of 343 wild boars from Northern Germany were first analyzed for anti-HEV IgG using an in-house ELISA, resulting in 19% positive samples. Subsequently, a subset of 41 representative samples was tested with the neutralization assay, and the results correlated well with those obtained by ELISA. Not only the human HEV strain 47832c but also two porcine HEV strains were shown to be neutralized by porcine serum antibodies. Neutralizing activity was also found in samples containing both HEV-specific antibodies and HEV RNA. Testing of serum samples derived from two experimentally infected domestic pigs showed a steep increase in neutralizing activity at 24 or 51 days post infection, dependent on the used infectious dose. The developed assay can be useful for characterization of the humoral immune response after HEV infection and for assessing the efficiency of HEV vaccine candidates.

## INTRODUCTION

A large variety of mammalian species including humans, pigs, deer, camels, bats, rats, and mongooses are susceptible to an infection with hepatitis E virus (HEV) (1). HEV is allocated to the family *Hepeviridae*, subfamily *Orthohepevirinae*, genus *Paslahepevirus*. Within the species *Paslahepevirus balayani*, eight different genotypes can be differentiated (2). Most of them can infect animal species; only HEV-1 and HEV-2 are restricted to humans and are transmitted through contaminated drinking water, especially in developing countries (3). The genotypes HEV-3, HEV-4, and HEV-7 are of zoonotic nature (4). They are distributed globally and are mostly transmitted to humans through the consumption of virus-containing animal products like meat, liver, or milk (5, 6). HEV-3 is the most prevalent genotype in Europe and is mainly found in domestic pigs and wild boar, but also in rabbits and deer (1).

In 2015, the World Health Organization estimated over 3.3 million symptomatic hepatitis E virus infections worldwide, leading to approximately 44,000 fatalities (7). In most cases, an infection with human restricted genotypes 1 and 2 leads to a self-limiting acute hepatitis with typical symptoms like fever, abdominal pain, and anorexia, but can progress to severe disease. Especially, pregnant women infected with HEV-1 are at high risk of mortality within the third trimester of pregnancy. Infections with the zoonotic genotypes can also lead to acute hepatitis but often remain asymptomatic. However, genotype 3 infections in immunocompromised individuals and especially in transplant patients can lead to chronic infection, which can develop to life-threatening liver cirrhosis. In addition, extrahepatic manifestations of HEV infection have been described (8).

The viral genome consists of a single-stranded, positive-sense RNA molecule with approximately 7.2 kb and encodes three open reading frames (ORFs), whereby the ORF1 encodes the non-structural proteins. The ORF3 protein, a small phosphoprotein, interacts with the icosahedral capsid encoded by the ORF2 and with components of the endosomal sorting complexes required for transport (ESCRT) machinery (9 – 11). For this reason, the ORF3 protein seems to be essential for formation of the viral quasi-envelope and may be the main driver for viral particle secretion (12). Quasi-enveloped HEV (eHEV) particles are covered by host-derived lipid membranes. Such particles can be found in serum of HEV-infected individuals, following viral release via the exosomal pathway at the basolateral side of hepatocytes, as well as in the cell culture supernatant of infected cells (9, 10, 12). In case the virus is released at the apical side into the bile canaliculi, the lipid membrane is removed by detergent effects of the bile acid, and subsequently, non-enveloped HEV (nHEV) particles pass the intestine and are shed into the environment (13). Several studies have shown that detergents like NP-40, sodium deoxycholate, or digitonin efficiently remove the quasi-envelope of eHEV (9, 14, 15). Both particle types are infectious in cell culture, but nHEV shows a higher infectivity as compared to eHEV (16).

Antibodies against HEV mainly bind to epitopes located in the C-terminal part (amino acids 423–606) of the ORF2 protein (17, 18). However, it is assumed that the lipid membrane protects eHEV particles from neutralizing antibodies by covering the viral capsid, a mechanism which can also be observed with other quasi-enveloped viruses like hepatitis A virus (9, 19).

The HEV antibody prevalence among German adults was approximated at 16.8% (20). In the course of an acute infection in human patients, an increase of anti-HEV IgM antibody concentration within the first weeks can be seen. Thereafter, IgM levels drop, and after 32 weeks, IgM is no longer detectable (21). On the contrary, anti-HEV IgG antibodies can be found over several years; one study reported a significant decrease of IgG levels after 5 years, while others demonstrated persistence of antibodies against HEV still 30 years after infection (22, 23). There are different assumptions up to which level IgG antibodies are protective against a subsequent re-infection with HEV, but there is no evidence for a lifelong immunity (23 – 25). The most common test to diagnose an HEV infection and to study the antibody status of patients is the enzyme-linked immunosorbent assay (ELISA) (25). However, the presence and function of neutralizing antibodies in humans and animals have been only scarcely analyzed so far, mainly due to the lack of suitable neutralization assays for HEV.

Concerning wild boar, the rate of HEV-seropositive animals in Germany exhibits large differences, depending on the hunting area. A progress study covering the years 2016–2020, for which the samples were collected in the South of Germany, revealed rates of antibody-positive wild boar sera between 7.5% and 14% (26). In another study, an increase in the detection rate of HEV-specific antibodies from 9.5% to 22.8% was determined between 2013 and 2017 in wild boars from Germany (27). The highest seroprevalence of 29.9% in wild boar was found in a study investigating 132 animals in 2007 (28). Also, the seroprevalence in domestic pigs in Germany was reported to be very high and ranged from 42.7% to 68.6% (29, 30).

In recent years, several cell culture systems have been developed for HEV propagation, although all of them are still laborious and time-consuming (31). In principle, they should also be suitable to analyze neutralizing activity of HEV-specific antibodies. However, the ability of antibodies to neutralize HEV and consequently to protect the host from infection was mainly tested through experimental infection of animals so far (24, 32).

The aim of this study was to evaluate the neutralizing capacity of HEV-specific antibodies in relation to their reactivity determined in ELISA. To this end, a novel cell culture-based neutralization assay was developed here. To characterize the assay, a subset of wild boar serum samples pretested by an IgG ELISA was analyzed for their neutralizing activity, and the results of both tests were compared. In addition, serum samples of experimentally HEV-infected domestic pigs were analyzed to monitor the development of neutralizing antibodies.

## MATERIALS AND METHODS

### Cell culture

The human hepatoma cell line PLC/PRF/5 (CLS cell lines service) was cultivated in Minimum Essential Medium Eagle supplemented with 10% heat-inactivated fetal calf serum (FCS), 2-mM L-glutamine, 1% non-essential amino acids, 100-U/mL penicillin G, and 100-µg/mL streptomycin (all reagents from PAN-Biotech, Aidenbach, Germany) at 37°C and 5% CO_2_. The cell monolayer was split in a ratio of 1:3–1:5 twice a week. Following virus infection, the medium was changed to Dulbecco’s Modified Eagle’s Medium (DMEM) (PAN-Biotech; 4.5-g/L glucose, without L-glutamine, without sodium pyruvate, 3.7 g/L NaHCO_3_) supplemented with 5% FCS and 2-mM L-glutamine (PAN-Biotech), and the plates were incubated at 34.5°C and 5% CO_2_.

### Virus and detergent treatment

For virus stock production, naïve PLC/PRF/5 cells were infected with human HEV genotype 3c strain 47832c, and the recently isolated porcine strains DP/spleen (HEV-3e) and WB/liver/21 (HEV-3, unassigned subgenotype) (33) for 1 hour at room temperature and subsequently incubated at 34.5°C for 3–4 weeks, with monitoring of viral RNA in the cell culture supernatant by an ORF3-specific reverse transcription-quantitative PCR (RT-qPCR) as described (34, 35). This assay had a detection limit of 188 genome equivalents/mL; negative and positive controls confirmed the validity of the RT-qPCR analyses (data not shown). Afterward, the cell culture supernatant was harvested, centrifuged for 10 min at 3,320 × *g*, and aliquots were stored at −80°C. To remove virus-associated lipids, the cell culture supernatant was treated with 1% sodium deoxycholate (DOC-Na) (Sigma) for 2 hours at 37°C on a shaker. Vivaspin 20, 50,000 MWCO PES Ultrafiltration Units (Sartorius, Göttingen, Germany) were used to remove the DOC-Na by centrifugation for 10 min at 3,320 × *g*. The flow-through was discarded, and the supernatant was exchanged with culture medium for a second centrifugation step under the same conditions. Subsequently, the remaining virus-containing supernatant was diluted with medium and stored at −80°C.

### Virus titration

To determine the viral titer, PLC/PRF/5 cells were seeded in a 96-well plate in a concentration of 1.04 × 10^4^ cells/cm^2^ and incubated for 2 weeks at 37°C and 5% CO_2_, while the medium was renewed twice a week. At day 14 after seeding, the cell culture medium was removed and the cell layer was washed once with 120 µL Dulbecco’s phosphate-buffered saline (DPBS) per well. Untreated virus and the DOC-Na treated virus was diluted in 10-fold series and tested in quadruplicates. For this, virus dilutions, prepared in a separate microtiter plate, were applied onto the cells and incubated for 1 hour at room temperature. After removing the inoculum, the cell layers were washed again once with 120 µL DPBS, and the wells were filled up with 120 µL DMEM. The medium was renewed completely after 3–4 days of incubation at 34.5°C and 5% CO_2_. At 7 days post infection (dpi), HEV capsid protein was stained with a rabbit anti-HEV hyperimmune serum (kindly provided by R. Ulrich, Friedrich-Loeffler-Institut, Greifswald, Insel Riems, Germany) and a polyclonal goat anti-rabbit IgG secondary antibody, and the plates were evaluated by fluorescence microscopy (33). For this, the focus-forming units (FFU) were counted in each well, and the viral titer was calculated based on the number of FFUs in the wells infected with the highest virus dilution showing specific HEV signals (36). One FFU was defined as one or more adjacent positive cells with a clear intracytoplasmic fluorescence signal.

### Serum samples

In total, 343 wild boar serum samples were collected in Northern Germany in the years 2017 and 2018 and stored at −80°C until they were investigated for the presence of HEV-specific antibodies by ELISA and neutralization assay as described below. Prior to the application in the neutralization assay, serum samples were inactivated at 56°C for 30 min. In addition, RT-qPCR analysis was performed for each sample, applying a previously described protocol (34). HEV RNA-positive sera were additionally treated at 70°C for 2 min to inactivate possibly contained virus, which could have distorted the determined infection rate; it has been reported that this treatment results in a reduction of at least 3.9 log_10_ FFU/mL (37). Serum samples from two experimentally HEV-infected domestic pigs were inactivated and analyzed in the same way like the wild boar serum samples. The pigs were infected with an HEV-3 strain derived from liver suspension of an experimentally infected wild boar, after they had been tested negative for HEV RNA in feces and serum. Antibodies against HEV were not detectable in serum through ELISA prior to infection. Blood and fecal samples were taken every 2–3 days during the whole investigation period (62 days for domestic pig 1 and 27 days for domestic pig 2). The animal experiments were approved by the responsible authority of the Federal State of Mecklenburg Western-Pomerania (38).

### ELISA

All wild boar serum samples were tested with a previously described in-house ELISA (39). Briefly, recombinant HEV-3 capsid protein was expressed in *Leishmania tarentolae*, purified, and coated on ELISA plates. After the serum samples were diluted 1:400, added onto the plates, and incubated for 1 hour at 37°C, a rabbit anti-pig IgG antibody, conjugated with peroxidase, was applied onto the wells to detect bound serum antibodies and incubated at 37°C for 1 hour as well. 3,3′,5,5′-tetramethylbenzidine served as the peroxidase substrate while incubating for 10 min in the dark. The optical density (OD) was measured at 450 nm. Sample-to-positive (s/p) ratio was calculated by subtracting the OD measured in the blank wells and subsequently dividing the OD of the sample by the OD of the positive control. Sera with an s/p value of <0.3 were considered as negative. Positive and negative controls (serum samples from domestic pigs) were previously confirmed through a commercially available test (ELISA PrioCHECK HEV Ab porcine, Prionics)

### Neutralization assay

Like in the protocol used for the virus titration, PLC/PRF/5 cells were seeded in 96-well plates and grown for 2 weeks. For this endpoint dilution method, for each serum sample, log_4_ dilution series from 1:4 to 1:16,384 (or from 1:4 to 1:67,108,864 for the highly reactive serum samples) in a total volume of 21 µL were prepared in a separate microtiter plate with DMEM without FCS. In total, 41 serum samples were available in sufficient amount and were tested in triplicates. While establishing the assay, a rabbit anti-HEV hyperimmune serum was used as a positive control. The DOC-Na treated virus was adjusted to a concentration of 1,143 FFU/mL (10^3.058^ FFU/mL) and added to each serum dilution (21 µL/well). After incubation of serum samples with the virus at 37°C for 1 hour on a shaker, the cells were washed once with 120 µL DPBS. Subsequently, 35 µL of the serum-virus inoculum, an equivalent to 20 FFU (10^1.3^ FFU), was applied onto the cells and incubated for 1 hour at room temperature. Using 20 FFU yielded the most stable results, and this infectious dose provided the best opportunity to count individual foci in each well (infectious doses of 10–100 FFU were tested). After this, the inoculum was removed, and the plate was washed again with DPBS. During the 1-week incubation period at 34.5°C and 5% CO_2_, the medium was renewed completely after 3 or 4 days. For each serum tested, two negative controls were included: one mock infected control well as well as a 1:10 dilution of the investigated serum lacking test virus.

At 7 dpi, the shortest period of time suited to analyze the infection rate, the plates were evaluated by immunofluorescence staining as described previously (33). Wells with less than six foci (<10^0.8^, a deviation of 0.5 log_10_ from 20 FFU) were evaluated as neutralizing antibody positive. In those wells, foci were very small, consisting of only 1–10 virus-positive cells. To calculate the neutralization titer [neutralization doses 50% (ND_50_), defined as reciprocal of the highest serum dilution where virus infection was inhibited in 50% of the wells], the Spearman-Kaerber formula was used (40). To prove the validity, in each run, the virus under test was titrated again in a twofold series dilution between 20 and 0.625 foci per well. All wells with at least one focus were included to calculate the mean value of foci per milliliter in each dilution step. The test was regarded as valid if the viral titer ranged between 361 and 3,614 FFU/mL (10^2.558^–10^3.558^ FFU/mL); for the experiments described in the present study, the viral titers ranged from 504.75 to 2,629.0 FFU/mL. Another criterion for validity was the absence of immunofluorescence signals of both controls (mock infected cells and cells inoculated with 1:10 serum dilutions lacking test virus).

## RESULTS

### Anti-HEV IgG antibodies in wild boar sera detected by ELISA

**Fig 1:**
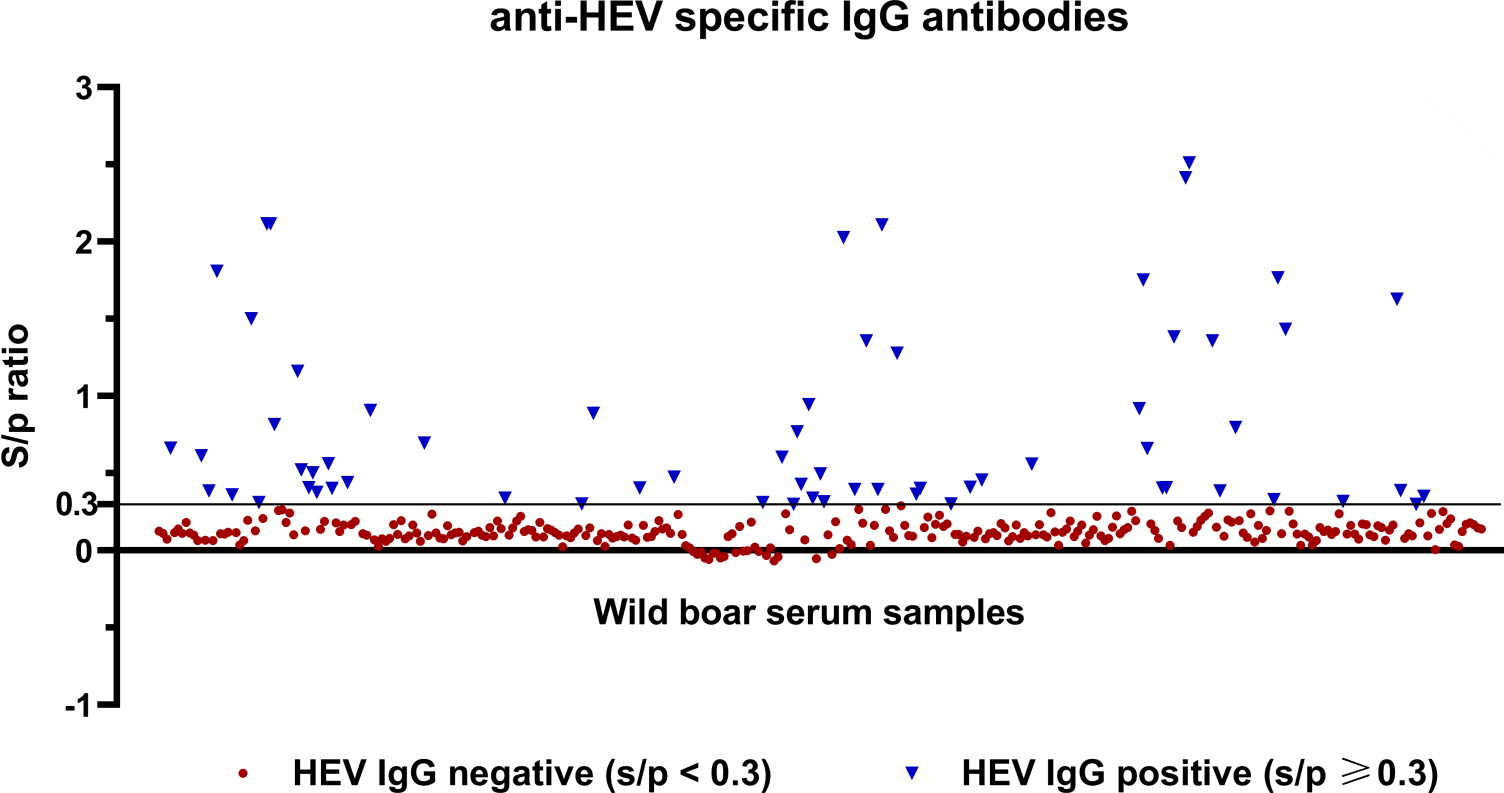
Distribution of s/p values of 343 wild boar sera, determined by in-house ELISA. Overall, 278 sera were tested negative for anti-HEV IgG antibodies, while 65 samples exhibited s/p values of ≥0.3 and were considered as antibody positive.

The presence of anti-HEV IgG antibodies in 343 wild boar serum samples was determined by an in-house ELISA. Optical densities obtained from sera under test as well as from one antibody-negative and one antibody-positive control serum were measured and a sample-to-positive ratio was calculated for each sample. 278 of the samples (81%) showed s/p values of <0.3 (ranging between −0.07 and 0.29) and were consequently defined as antibody-negative. In turn, 65 of the sera (19%) revealed s/p values of ≥0.3 within a range of 0.3–2.51. Conclusively, those were considered as being positive for anti-HEV IgG-specific antibodies (Fig. 1).

### Comparative neutralization of untreated and DOC-Na-treated virus

**Fig 2:**
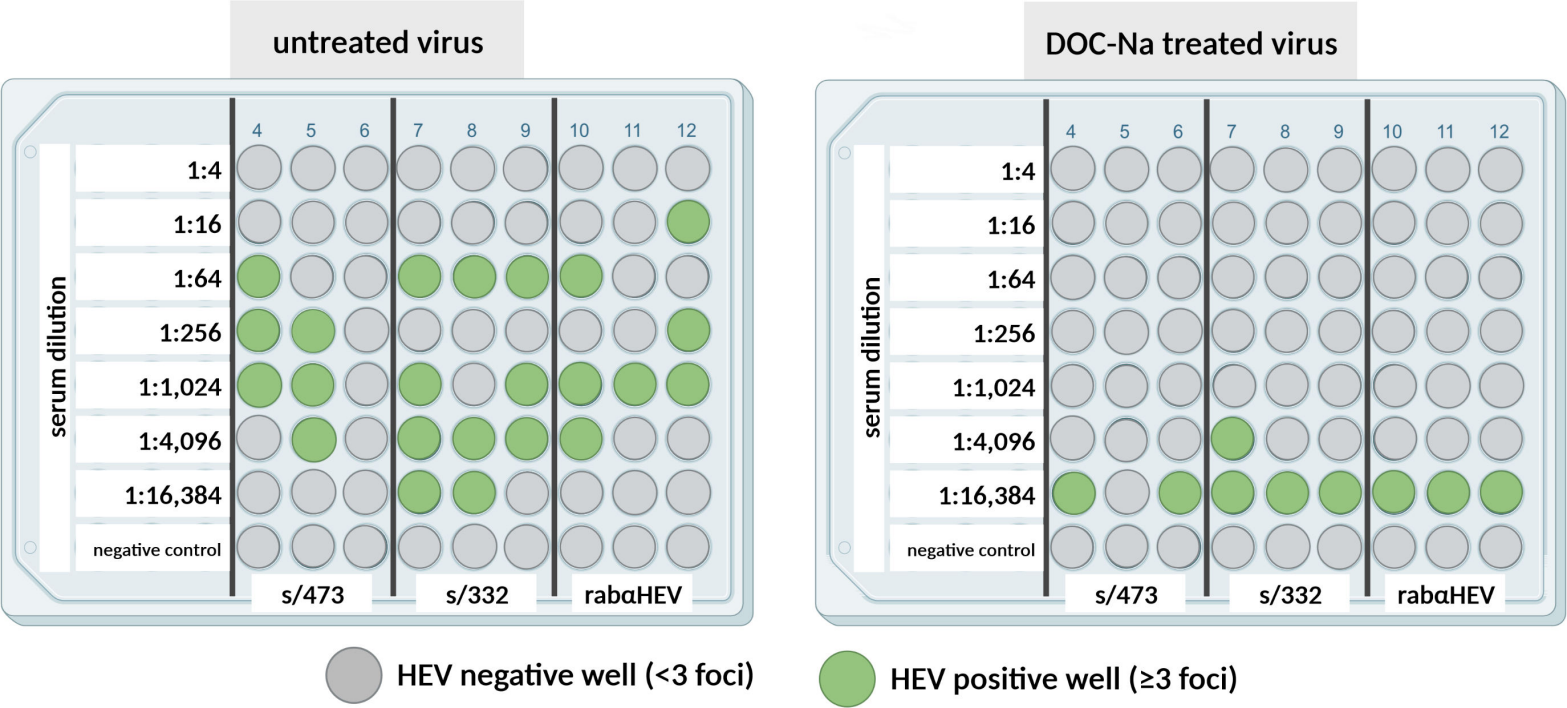
Neutralization of either untreated (left) or DOC-Na-treated (right) HEV by three different anti-HEV antibody-positive sera (s/473, s/332, and rabαHEV). Green dots indicate HEV-positive wells with ≥3 fluorescence foci; gray dots indicate HEV-negative wells with <3 fluorescence foci. Created with BioRender.com

To characterize the neutralizing properties of antibodies present in the wild boar serum samples, a cell culture-based endpoint dilution neutralization assay was established, which used immunofluorescence staining as readout of virus infection. The neutralization of untreated HEV and virus pretreated with bile acid to remove the quasi-envelope was compared. Three antibody-positive sera as determined by ELISA were selected for this: wild boar sera s/473 (s/p value = 1.63, strongly positive) and s/332 (s/p value = 0.4, weakly positive) and the rabbit hyperimmune serum. To compare neutralization of both virus forms, contrary to the standard protocol established later, 10 FFU (10^1^) were applied to each well, and consequently, wells with at least three fluorescence foci (10^0.5^) were evaluated as HEV-positive. Figure 2 shows the schematic illustration of the results after immunofluorescence analysis. None of the three tested sera did clearly neutralize the untreated virus; instead, arbitrarily distributed positive wells were observed. In contrast, clear patterns were evident for the DOC-Na-treated virus, which allowed the determination of neutralization titers (ND_50_) of 5,082 for serum s/332 and 8,128 for the rabbit hyperimmune serum. The neutralization titer of serum s/473 exceeded 8,128 but could not be exactly determined due to the lack of higher serum dilutions (Fig. 2).

### Neutralizing capacities of anti-HEV-specific antibodies in wild boar serum samples

**Fig 3:**
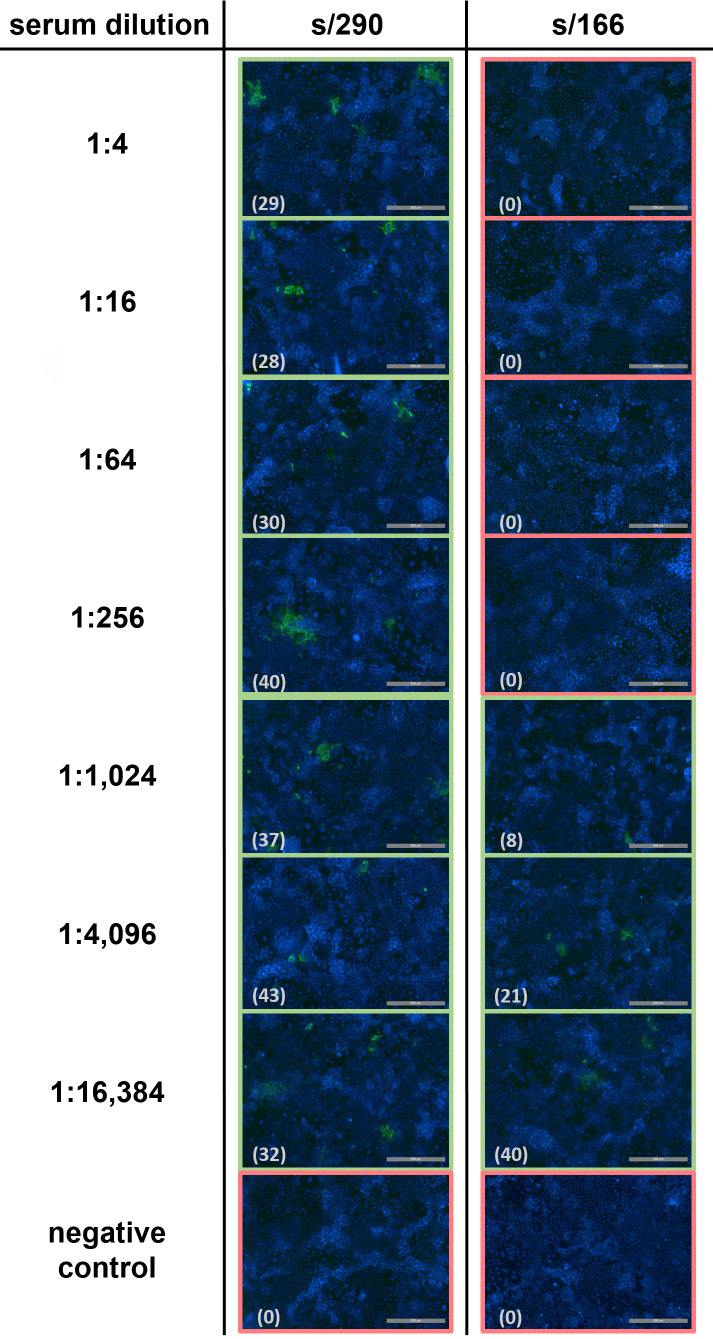
Illustration of immunofluorescence staining following virus neutralization of wild boar sera s/290 (s/p ratio of −0.005) and s/166 (s/p ratio of 1.808). Shown are exemplary image details of PLC/PRF/5 cells infected with virus-serum mixture containing serum dilutions between 1:4 and 1:16,384 (analyses were performed in triplicates; one row is depicted here). The total number of foci in each well is given in brackets for each image. HEV-positive wells (≥6 foci) are framed in green; wells determined as HEV negative (<6 foci) are framed in red. ND_50_ values were determined as ≤4 for S/290 and 805 for s/166, respectively. HEV-specific foci = green; nuclei = blue; scale bar 500 µm.

In total, 41 HEV RNA-negative wild boar sera with corresponding ELISA s/p values between −0.05 and 2.51 were selected and analyzed for their properties to neutralize DOC-Na-treated HEV: 13 samples had an s/p ratio of <0.3; 17 ranged between 0.3 and 1.0; and 11 sera were above an s/p value of 1.0. As a positive control for the neutralization assay, the rabbit anti-HEV hyperimmune serum was utilized, and a neutralization dose ND_50_ of 5,129 was determined by immunofluorescence analysis. The immunofluorescence staining following neutralization with two sera (s/290 and s/166) is exemplarily shown in Fig. 3.

**Fig 4:**
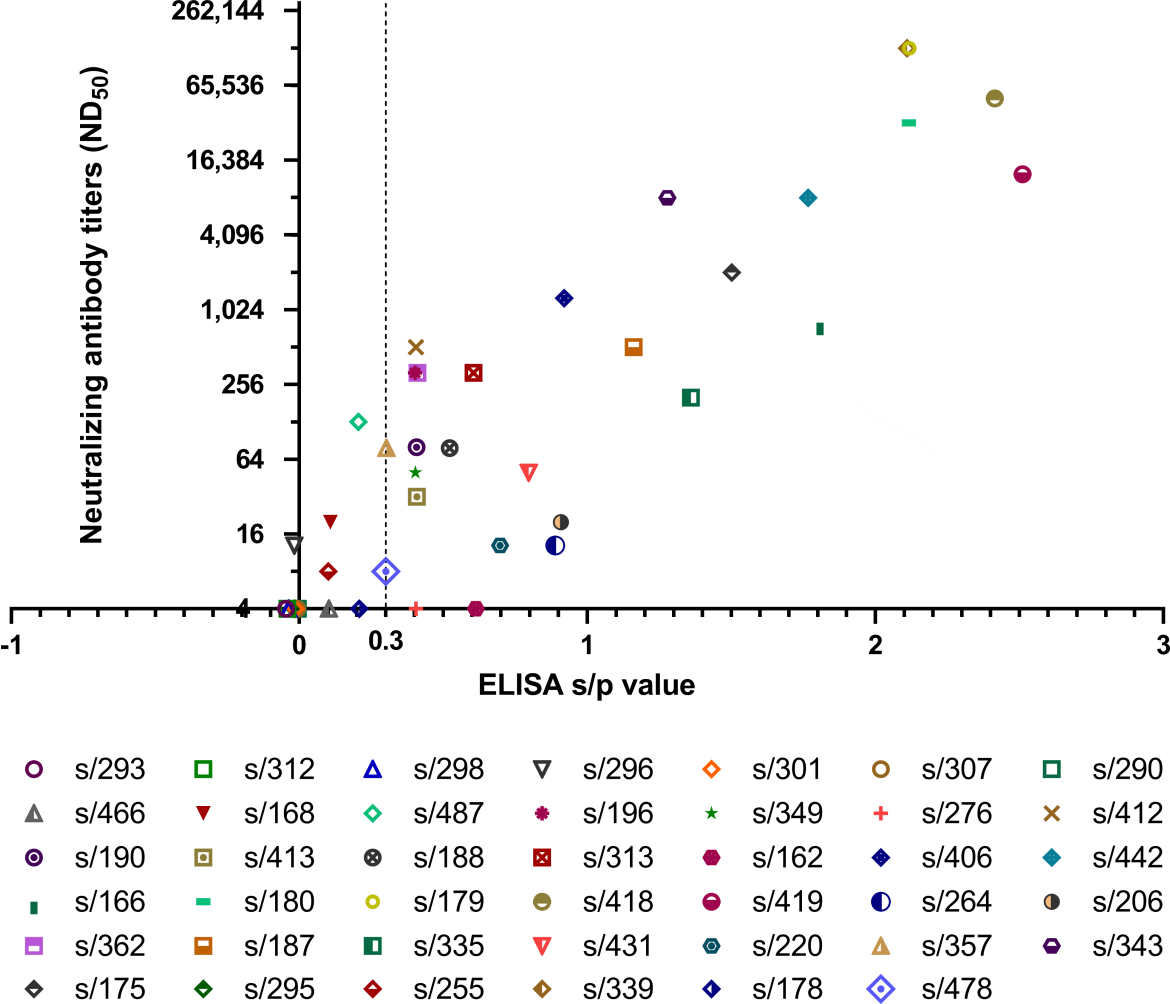
Neutralizing antibody titers (ND_50_) of *n* = 41 wild boar serum samples in relation to corresponding s/p values determined by ELISA. The ELISA cut-off value (0.3) is indicated by a dashed line.

Generally, the ND_50_ of the wild boar sera corresponded well with s/p ratios determined by antibody ELISA (Fig. 4). Serum samples s/179 and s/339 exhibited ELISA s/p ratios of 2.12 and 2.11, respectively. In both sera, the highest neutralization titer among the investigated samples was measured (both: ND_50_ = 130,617). Similar results were obtained for sera s/180 (s/p value = 2.12, ND_50_ = 32,659), s/418 (s/p value = 2.41, ND_50_ = 51,523), and s/419 (s/p value = 2.51, ND_50_ = 12,589). Nine sera showed no neutralization (titer ≤4). In four samples with an s/p ratio of <0.3, low neutralization titers between 8 and 128 were detected. In sera with s/p values between 0.30 and 0.91, ND_50_ titers ranged from 8 to 511. Although ELISA s/p values of the sera s/162 and s/276 were ≥0.3, no neutralizing antibodies could be determined for these samples.

### Neutralization capacities of HEV RNA-positive wild boar sera

In total, 12 of 343 sera were tested positive for both anti-HEV antibodies (analyzed by ELISA) and HEV RNA, of which 10 were available in sufficient amount and applied in the neutralization assay after an additional virus inactivation step at 70°C. ELISA s/p values of those sera ranged from 0.3 (s/316) to 1.38 (s/415). The ND_50_ of serum s/317 could not be determined because the sample had clotted after inactivation. Eight serum samples showed moderate to high ND_50_ titers ranging from 201 (s/324) to 32,360 (s/195), while a titer of only 20 was determined for the remaining serum sample (s/474). The RT-qPCR analysis revealed Cq values between 31.76 and 37.81 for these sera (Table 1). In a previous study, HEV-3 strains were isolated from three corresponding liver samples (s/415, s/425, and s/474), and additional 15 strains from the respective wild boar cohort were assigned to genotype 3 as well (33).

**TABLE 1:** Cq values (determined by RT-qPCR), ELISA s/p values, and neutralization titers (ND_50_) of HEV RNA-positive wild boar sera

Sample	Cq value	s/p value ELISA	ND_50_
s/318	31.76	0.49	8,166
s/425	31.81	1.36	805
s/474	32.03	0.39	20
s/415	32.08	1.38	12,882
s/195	33.24	0.56	32,360
s/317	34.92	0.77	Not tested
s/323	35.17	0.50	2,042
s/324	36.59	0.32	201
s/320	36.62	0.95	318
s/316	37.81	0.30	511

### Neutralization capacities of serum antibodies in samples from domestic pigs after experimental HEV infection

**Fig 5:**
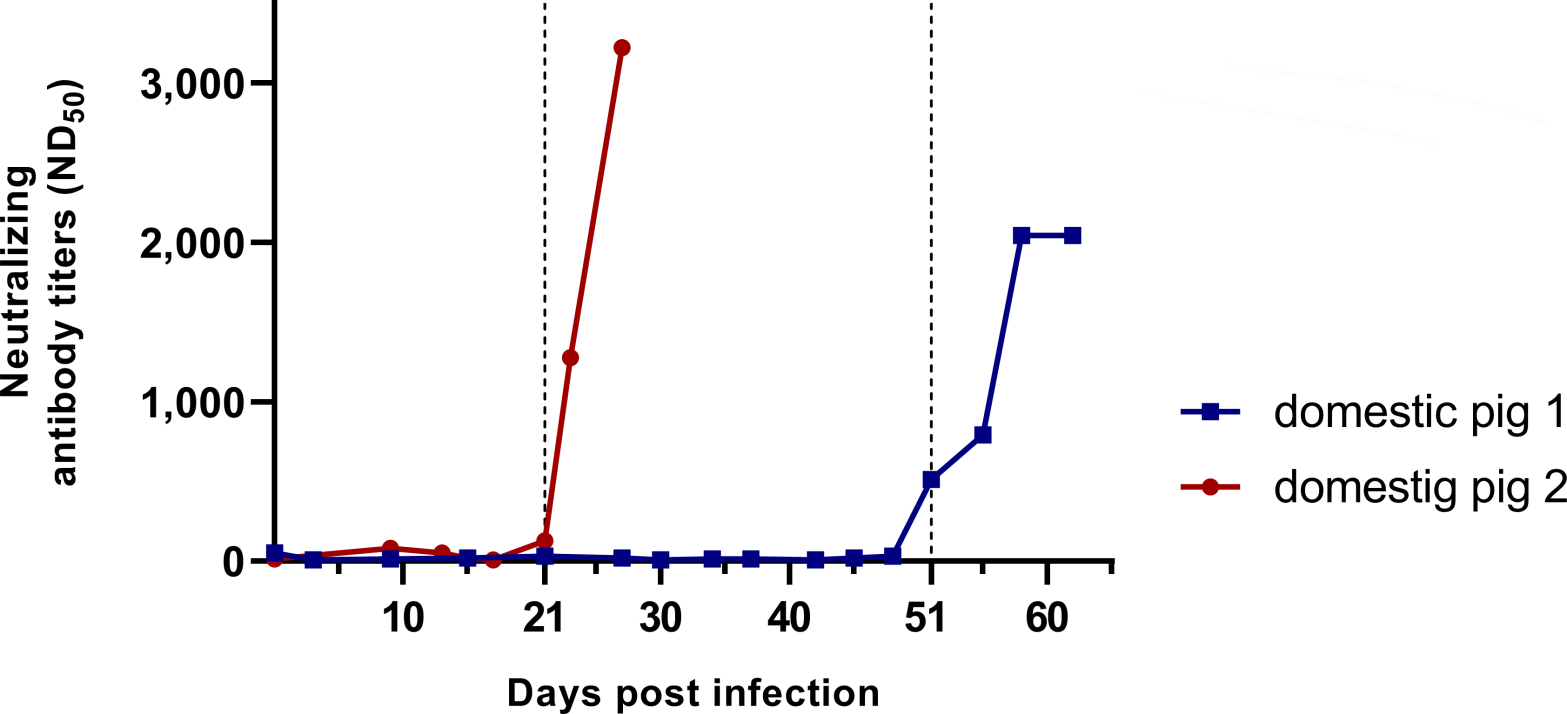
Neutralizing antibody titers (ND_50_) in serum samples from two domestic pigs after experimental HEV infection. Day 21 (domestic pig 2) and day 51 (domestic pig 1) after infection are marked with dashed lines indicating seroconversion detected by ELISA (38).

During an experimental infection of domestic pigs with HEV genotype 3, 16 serum samples were collected over 62 days from animal 1, whereas 9 samples were collected over 27 days from animal 2. The inoculum which was used to infect animal 1 contained 65.1 HEV RNA copies/dose and that for animal 2, it contained 935,706.8 HEV RNA copies/dose (38). The serological status was determined by a commercially available species-independent ELISA detecting IgG, IgM, and IgA (AXIOM, Bürstadt, Germany), and seroconversion was demonstrated at 51 dpi (animal 1) and at 21 dpi (animal 2), respectively (38). To evaluate the presence of neutralizing antibodies in these sera, the neutralization assay reported in this study was applied. At the time of seroconversion, a neutralization titer of ND_50_ = 511 was determined for animal 1 (at 51 dpi) and an ND_50_ of 127 was found for animal 2 (at 21 dpi). The neutralization titers increased for animal 1 during the following 11 days to ND_50_ = 2,042 and for animal 2 to ND_50_ = 3,221 at 27 dpi, respectively (Fig. 5). Prior to seroconversion, the neutralizing antibody titers (ND_50_) for animal 1 were ≤8 (days 3, 30, and 42 after infection), while analysis of the sample collected at day 0 revealed a titer of ND_50_ = 50. The neutralization titers for sera collected from animal 2 before 21 dpi ranged from 8 to 79 (ND_50_ day 0 = 13, ND_50_ day 9 = 79, ND_50_ day 13 = 50, ND_50_ day 15 = 20, and ND_50_ day 17 = 8). The serum sample of animal 2 taken at 6 dpi was not applicable in the neutralization assay due to clotting after inactivation.

### Neutralization of porcine HEV strains

**Fig 6:**
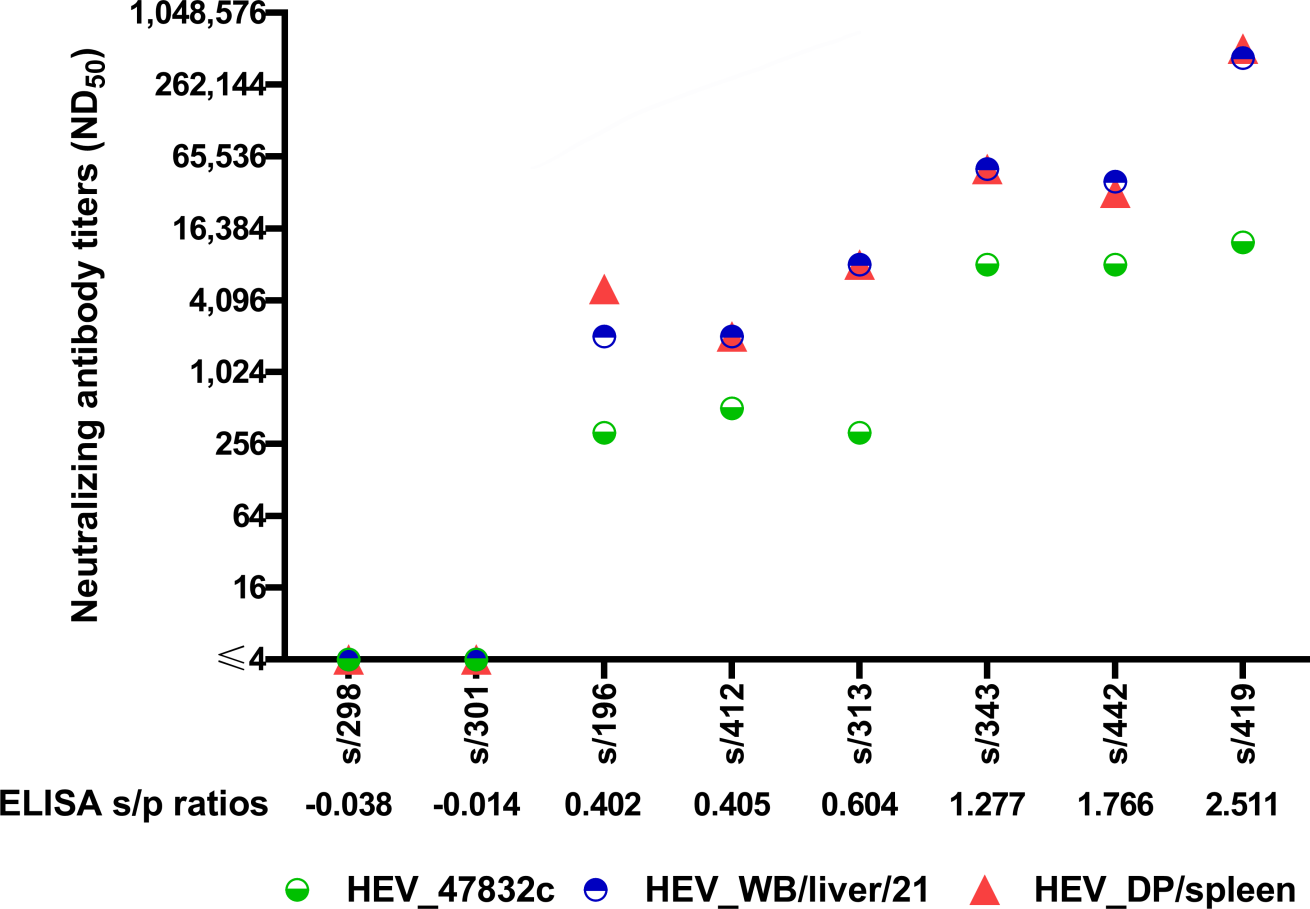
Neutralizing antibody titers (ND_50_) of eight wild boar serum samples determined with three different HEV strains: 47832c (green), WB/liver/21 (blue), and DP/spleen (red). Serum samples are sorted by ascending ELISA s/p values, left to right.

A total of eight wild boar serum samples with corresponding ELISA s/p ratios from −0.038 (s/298) to 2.511 (s/419) were additionally analyzed in the neutralization assay with two porcine HEV field strains: the HEV-3e strain DP/spleen and the strain WB/liver/21 (unassigned HEV-3 subtype) (33). The ELISA negative samples s/298 and s/301, which could not neutralize the human strain 47832c also failed to neutralize both viruses of porcine origin. The remaining six sera yielded considerably higher neutralization titers after application of porcine HEV as test virus compared to the human strain. Results differ by factors of 4.0–41.7 (Fig. 6).

## DISCUSSION

Since the discovery of the hepatitis E virus in 1983 and the description of the first full-length sequence in 1991, several attempts have been made to determine neutralizing properties of anti-HEV-specific antibodies (41 – 43). An early study from 1997 described a PCR-based serum neutralization assay analyzing HEV RNA in cells after inoculation with a mixture of virus and monkey sera from an experimental HEV infection, but the assay has not been widely used further (44). Further neutralization assays have been developed using cell culture- or stool-derived viral particles from infected human patients or experimentally infected monkeys (42, 45). However, the availability of stool samples from HEV-shedding individuals was the limiting factor of these assays. An alternative approach to determine the neutralizing capacity of serum antibodies includes the use of virus-like particles, which were labeled with a fluorophore. The neutralization titers determined by this method showed good correlations with the amount of anti-HEV IgG antibodies measured by ELISA, although a comparison with neutralization of infectious virus was not shown (41). Very recently, HEV strain KernowC1/p6 was incubated with sera from experimentally infected monkeys prior to inoculation onto permanent cells. Subsequently, secreted ORF2 protein was measured by ELISA as an indicator for successful virus infection of cells (46). Taken together, although several different assays for detection of monkey or human HEV-neutralizing antibodies have been described, none of these assays has been shown to be suitable for routine analysis of larger numbers of samples. Moreover, wild boar and domestic pigs as the most important animal reservoir hosts have not been studied so far.

In our study, we established a cell culture-based neutralization assay to address this issue. The well-established cell culture-adapted human HEV genotype 3c strain 47832c (35, 47, 48) was used in combination with the human hepatoma cell line PLC/PRF/5, which has been previously shown to efficiently support HEV replication (33, 49, 50). Additionally, a subset of serum samples were tested by using the recently isolated porcine HEV-3 field strains WB/liver/21 and DP/spleen (33). In our assay, the usage of a 96-well format enabled the quantification of neutralizing antibodies for a large number of samples, and the direct visualization of viral antigen by immunofluorescence allowed a reliable identification of infection and virus replication.

In our first experiments, we could show that treatment of HEV with detergent was necessary to obtain reliable results by the neutralization assay. This is in line with previous studies showing that removal of the lipid membrane from eHEV particles was mandatory to enable antibody binding (9, 10). Our results support the finding that lipid-associated HEV particles as present in the bloodstream or cell culture supernatant cannot be directly neutralized by antibodies, since their epitopes are shielded by the quasi-envelope of these particles. In contrast, after removal of the envelope by DOC-Na treatment, the neutralization was readily shown in our assay, indicating that the epitopes of the HEV particles targeted by neutralizing antibodies were accessible now. Lower cell-binding efficiency of eHEV compared to nHEV as well as different binding and infection kinetics of both virus forms combined with a short incubation time of 1 hour might have contributed to divergent results obtained with the DOC-Na-treated and untreated HEV (16). Moreover, it can be hypothesized that the incomplete neutralization of eHEV is caused by the competence of IgG antibodies to bind viruses in the intracellular compartment. For IgG immunoglobulins, the potential to mediate intracellular neutralization has been described in infections with, for example, influenza A virus and rotavirus after binding of the virus to the host cell (51 – 53). Introduction of antibodies into the intracellular compartment prior to infection with virus might have been necessary to achieve complete intracellular neutralization.

Prior to the neutralization assay, the used sera were screened by ELISA for the presence of antibodies. By this, 19% of 343 wild boar sera were shown to be anti-HEV IgG positive, indicating a moderate to high HEV prevalence in wild boar populations in Lower Saxony, which corresponds to previous studies (26 – 28).

To validate the newly established neutralization assay, 41 serum samples with differing ELISA s/p values were selected to evaluate the presence of virus-neutralizing antibodies. For the great majority of the investigated samples, their neutralizing capacity correlated very well with the antibody level determined by ELISA. Only two of these sera showed no specific neutralization reaction, but their ELISA s/p values were rather low (s/162: 0.61 and s/276: 0.41). On the one hand, this could be due to the fact that not all antibodies have neutralizing properties but are rather responsible for detecting and labeling and might be involved in opsonization of pathogens (54). On the other hand, the neutralization assay might show a higher specificity compared to ELISA, and the samples were determined false positive in the initial analysis.

In contrast, four wild boar sera which were negative for specific anti-HEV IgG antibodies in the ELISA assay showed low HEV neutralization titers. One reason might be that the ELISA detected only IgG, but neutralizing functions are also known for IgM and IgA antibody classes (54, 55). Especially IgM antibodies are indicative for the early acute phase of an infection, while IgG is not yet detectable at this time point. Thus, the observed neutralizing effects might have been caused by IgM antibodies. Alternatively, the observed discrepancy could also be explained by a higher sensitivity of the neutralization assay compared to ELISA.

Interestingly, neutralizing antibody titers from 20 (s/474) to 32,360 (s/195) were also found for sera, which simultaneously contained HEV RNA. On the one hand, an incipient antibody response during viremia at an early stage of acute infection may explain these findings. On the other hand, the presence of HEV RNA and neutralizing antibodies at the same time could be indicative for insufficient viral clearance due to HEV immune evasion. One possible mechanism implicated in HEV persistence could be the presence of eHEV in the bloodstream, which cannot be directly neutralized as discussed above. In addition, the recently described production of another ORF2 protein species functioning as decoy may lead to a substantial binding of neutralizing antibodies to these antigens, which are no longer available to neutralize infectious virus (56 – 58). The lack of HEV neutralization was also observed in a chronically infected wild boar that shed virus over months, although high antibody titers were detected by the ELISA (59). Generally, it has been reported that the T-cell response plays an important role in controlling HEV infection (60, 61). However, to what extent the humoral immune response contributes to the process of viral clearance and the establishment of possible persistent infections calls for further investigations. Especially with regard to the protective function of the quasi-envelope, the importance of *in vivo* nHEV neutralization needs to be clarified in future studies.

Using our assay, we found that neutralizing properties could also be demonstrated in serum samples from two experimentally HEV-infected domestic pigs. At 21 dpi (animal 2) and 51 dpi (animal 1), anti-HEV antibodies were detected by a commercially available ELISA (38). At the same time points, neutralization titers of ND_50_ = 127 and 511 were determined, which increased during the subsequent sampling periods. This indicates that efficient neutralizing antibody activity starts at the same time after infection of pigs as the first antibodies can be detected by ELISA. Interestingly, production kinetics of neutralizing antibodies were dependent on the inoculated virus dose; after infection with a higher dose of HEV, the observed neutralizing antibody response was earlier and stronger. At time points prior to seroconversion, only low neutralization titers were detected (ranging from 8 to 50 for animal 1 and from 8 to 79 for animal 2), which might reflect unspecific reactions. Consequently, a cutoff might be set individually for each domestic pig, which would assess sera with ND_50_ of ≤50 for animal 1 and ND_50_ of ≤79 for animal 2 as negative concerning neutralizing antibodies. However, analyses of more samples from domestic pigs are necessary to determine a reliable cutoff for definition of neutralization positivity. We also demonstrated neutralizing activity of a rabbit control serum that was generated by immunization with a truncated HEV capsid protein expressed in *Escherichia coli* (62). This result shows that the capsid protein is able to induce neutralizing antibodies, which is in line with the results of the only commercially available HEV vaccine HEV-239 based on *E. coli*-expressed capsid protein (63).

This study is based on the use of the human HEV genotype 3c strain 47832c. To verify our results and demonstrate the robustness of this assay, two recently isolated HEV strains of porcine origin were applied as test virus. For all sera analyzed, the neutralization titers determined with the porcine HEV strains were 4.0- to 41.7-fold higher compared with the neutralization of the human HEV strain. Although the (sub-)genotypes of the HEV field viruses responsible for the production of antibodies present in the samples analyzed here are not known, it is reasonable to speculate that the differences in neutralization titers are due to a different degree of genetic/antigenic relatedness between the field viruses and the three test viruses used.

Taken together, this study affirmed a moderate to high prevalence of hepatitis E virus-specific antibodies in wild boar in Northern Germany. The main focus of this study was the development of a cell culture-based assay to measure neutralizing properties of anti-HEV antibodies in porcine serum samples. The results proved that the vast majority of ELISA-positive samples also contain virus-neutralizing antibodies. The efficient interspecies cross-neutralization of a human HEV-3 strain highlights the zoonotic potential of HEV genotype 3, while higher neutralizing capacities measured with two porcine test viruses call for further investigations. Moreover, the established cell culture-based neutralization assay will allow detailed studies of the humoral immune response during acute and persistent HEV infections in human patients and animal hosts and can be used for the efficiency assessment of HEV vaccine candidates.
